# Intracerebroventricular administration of chondroitinase ABC reduces acute edema after traumatic brain injury in mice

**DOI:** 10.1186/s13104-016-1968-8

**Published:** 2016-03-12

**Authors:** John D. Finan, Frances S. Cho, Steven G. Kernie, Barclay Morrison

**Affiliations:** Department of Neurosurgery, NorthShore University Health System, 1001 University Place, Evanston, IL 60201 USA; Department of Biomedical Engineering, Columbia University, 351 Engineering Terrace, Mail Code 8904, 1210 Amsterdam Avenue, New York, NY 10027 USA; Department of Pediatrics and Pathology and Cell Biology, Columbia University Medical Center, 3959 Broadway, CHN 10-24, New York, NY 10032 USA

**Keywords:** Traumatic brain injury, Edema, Chondroitinase ABC, Therapy

## Abstract

**Background:**

Brain edema is a significant challenge facing clinicians managing severe traumatic brain injury (TBI) in the acute period. If edema reaches a critical point, it leads to runaway intracranial hypertension that, in turn, leads to severe morbidity or death if left untreated. Clinical data on the efficacy of standard interventions is mixed. The goal of this study was to validate a novel therapeutic strategy for reducing post-traumatic brain edema in a mouse model. Prior in vitro work reported that the brain swells due to coupled electrostatic and osmotic forces generated by large, negatively charged, immobile molecules in the matrix that comprises brain tissue. Chondroitinase ABC (ChABC) digests chondroitin sulfate proteoglycan, a molecule that contributes to this negative charge. Therefore, we administered ChABC by intracerebroventricular (ICV) injection after controlled cortical impact TBI in the mouse and measured associated changes in edema.

**Results:**

Almost half of the edema induced by injury was eliminated by ChABC treatment.

**Conclusions:**

ICV administration of ChABC may be a novel and effective method of treating post-traumatic brain edema in the acute period.

## Background

Traumatic brain injury (TBI) is responsible for 36 % of deaths among children 1–14 years old and is a leading cause of death among those in the 15–45 year age-bracket [1]. It is also responsible for 40 % of combat deaths among US military personnel [2]. Brain edema within the rigid confines of the skull elevates intracranial pressure (ICP) [3] and, in severe cases, leads to herniation of the brain tissue which is frequently fatal [4]. Elevated ICP occludes blood vessels causing cerebral hypoperfusion, and elevated ICP correlates with poor outcome [5]. Unfortunately, currently available tools for managing post-traumatic edema suffer from serious limitations. Draining cerebrospinal fluid (CSF) compensates for the increased volume of the brain to a point, but the volume of CSF available to drain is often less than that needed to accommodate severe edema. Hyper-osmotic solutions of saline or mannitol can be administered systemically to draw water out of the brain. However, the subsequent reduction of ICP is sometimes short-lived and may be followed by rebound of ICP to or above the pre-treatment level [6]. Some surgeons perform decompressive craniectomies to relieve ICP in refractory cases. Clinical trial data on the effectiveness of this intervention is currently lacking [7], and the risk of infection and injury to the brain at the edge of the craniectomy are important drawbacks [8]. In summary, there is an urgent clinical need for new therapeutic strategies to address post-traumatic brain edema.

Coupled electrostatic and osmotic forces cause edema in injured brain tissue [9, 10]. In brain, as in many biological tissues, an ionic solution permeates a porous, solid matrix containing large, immobile, negatively charged molecules. The negatively charged molecules that are bound to the solid phase of the tissue electrostatically attract ions from the intracellular and extracellular fluid, thereby increasing the ion-concentration within the tissue as compared to the fluid surrounding the tissue and creating an osmotic gradient that draws in water. A field of biomechanics known as triphasic theory quantitatively describes these coupled electrostatic and osmotic forces [11]. Previously we have shown that biologically inert (i.e. dead) brain tissue swells in a manner that conforms to the highly non-linear predictions of triphasic theory, validating this model [9]. The healthy brain is highly metabolic, and much of the energy generated by metabolism is used to regulate ion concentrations in the tissue [12]. Therefore, healthy brain tissue is normally far from thermodynamic equilibrium, and its volume is less than that predicted by the physical laws of triphasic theory. However, when the brain is injured and ion flux out of cells is compromised, either because of reduced metabolism or damaged cell membranes, the damaged tissue moves toward thermodynamic equilibrium, i.e. it swells.

This physical model of post-traumatic brain edema motivated our therapeutic strategy. One of the most abundant of the large, immobile, negatively charged molecules in the tissue that drive edema is chondroitin sulfate proteoglycan (CSPG) [9]. CSPG also has a very low pKa (between 3 and 4 depending on the ionic strength of the solution [13]), meaning that it is very negatively charged at physiological pH. Chondroitinase ABC (ChABC) is an enzyme that degrades CSPG, thereby mobilizing its negative charge. This allows it to diffuse out of the solid matrix and reduce the osmotic potential to draw in water. ChABC has been studied at length in the central nervous system as a means of breaking down glial scars that inhibit axon regeneration [14–16] and is generally well-tolerated in rodents. For example, there was no change in visual field or acuity after ChABC was injected into the visual cortex in mice [17]. In addition to being generated in response to injury during scar formation, CSPGs exist throughout the brain in the absence of injury. They are abundant in perineuronal nets, extracellular structures that surround neurons and inhibit synaptic plasticity [18]. Previous in vitro work by our group showed that ChABC reduced edema in dead and injured brain tissue [10]. Therefore, we hypothesized that administration of ChABC would reduce post-traumatic brain edema in the mouse model of controlled cortical impact (CCI) TBI.

## Methods

All animal procedures were conducted according to the guidelines set forth in the *Guide for the Care and Use of Laboratory Animals* [19] and were approved by the Institutional Animal Care and Use Committee at Columbia University. A total of 40 mice were used in this study. Ten week old male C57/BL6 mice were anesthetized with isoflurane and placed in a stereotactic frame. The skull was exposed, and a 5 mm diameter craniotomy was drilled to the left of the sagittal suture between lambda and bregma. An Impact One device (Leica Biosystems, Buffalo Grove, IL) was used to propel a 3 mm diameter, flat-ended, cylindrical indenter into the brain to a depth of 1.3 mm at a speed of 5 ms^−1^ with a dwell time of 300 ms at a 10° angle to the vertical. In uninjured controls, the skull was exposed but not opened to eliminate the risk of damage during the craniotomy. The treatment consisted of 10 μl of a filter-sterilized 50 U/ml solution of ChABC (Sigma Aldrich, St. Louis, MO) dissolved in PBS. Within 5 min after injury, ChABC or vehicle was administered to the right lateral ventricle using a Hamilton syringe with a 26G needle inserted to a depth of 3 mm at a location 1 mm to the right and 0.3 mm anterior to bregma [20]. In preliminary experiments, a green tracer dye was injected to verify that agents injected at this point permeated the ventricular network. A group of uninjured animals was also injected to measure the effect of ChABC on water content in healthy brain tissue. To evaluate the effect of treatment 5 min after injury on edema 24 h after injury, animals were euthanized by cervical dislocation under deep anesthesia at the 24 h time point and the brains were immediately removed. A 4 mm thick coronal slice of brain tissue encompassing the lesion site was cut from the mid-brain and split into two hemispheres (ipsilateral and contralateral to the injury). Each hemisphere was weighed, dried at 95° C for 72 h, and then weighed again to determine the water fraction.

## Results and discussion

**Fig. 1:**
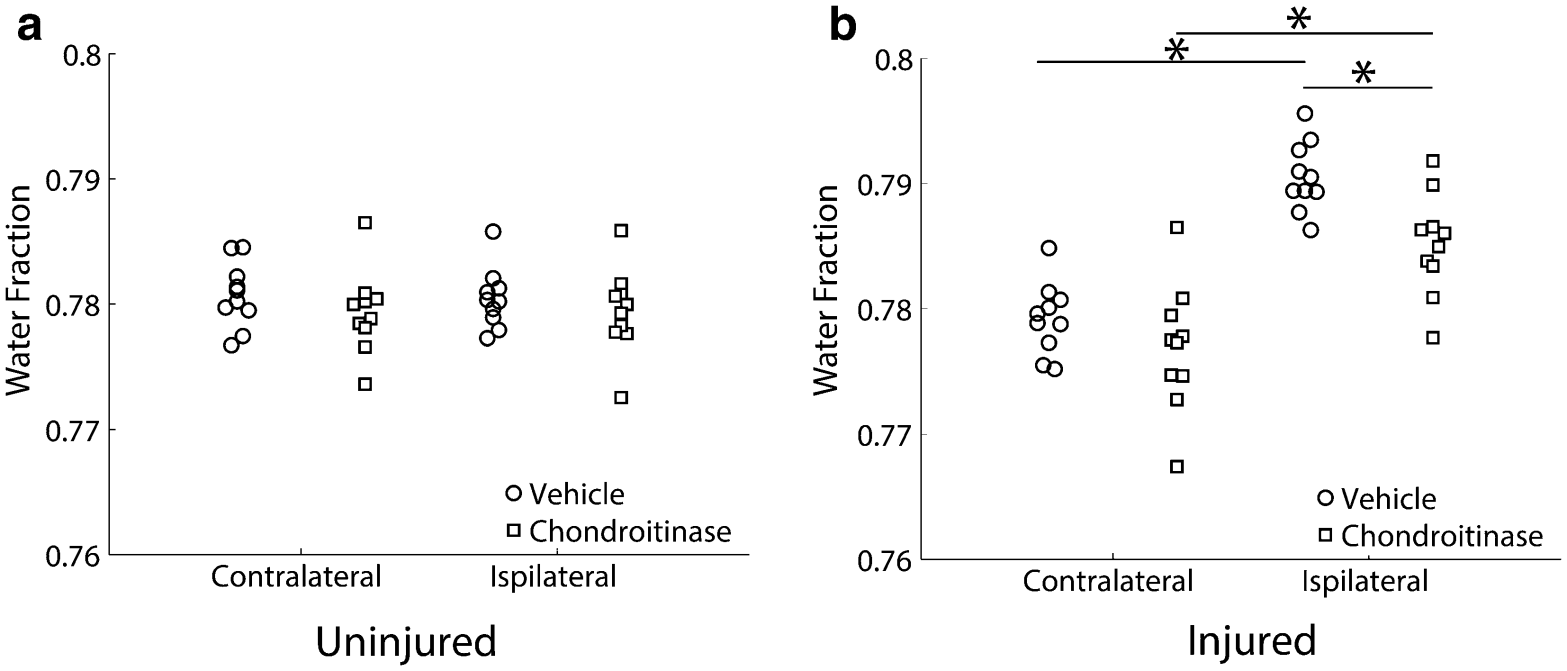
The effect of ChABC treatment on brain water content. **a** Water fraction was not significantly affected by ChABC treatment in uninjured animals. **b** ChABC treatment reduced water fraction in the brain hemisphere ipsilateral to the injury in injured animals (*, p < 0.05, ANOVA followed by Bonferroni post hoc test, mean ± standard error, n = 10)

There was no statistically significant effect of ChABC treatment on water fraction in uninjured animals (n = 10, Fig. 1a). In injured animals, the water fraction was 1.13 % higher in the ipsilateral hemisphere than in the contralateral hemisphere, indicating edema due to injury (Fig. 1b). Previously reported values for edema in this injury model range from 1 to 3 % [21–24]. These values are small because controlled cortical impact induces a focal lesion so only a small domain in the tissue sample tested has increased water content. Smaller tissue samples excised at the lesion site in a controlled cortical impact model showed a greater change in water content [25]. Treatment significantly reduced ipsilateral water fraction by 0.54 % (p < 0.05, ANOVA followed by Bonferroni post hoc test, n = 10, Fig. 1b), indicating that almost half of the edema induced by trauma was eliminated. The fact that the treatment affected water fraction more in injured animals than in uninjured animals suggests that it acts selectively on injured tissue. This selectivity may be an important advantage in light of the global delivery modality tested here.

There are several clinically established treatments for brain edema, and others have been proposed. However, none of the established or proposed treatments employs the same mechanism of action as ChABC treatment, suggesting that ChABC treatment would offer additional benefits when applied in combination with other established or proposed treatments for brain edema. Established treatments such as elevation of the head, CSF drainage, hyperosmotic therapy and decompressive craniectomy do not alter the fixed charge density, which is the target of ChABC treatment. Inhibition of aquaporin 4 (AQP4) has been proposed as a treatment for brain edema [26]. Aquaporin 4 is a water channel in the blood brain barrier (BBB). Genetic deletion of AQP4 reduced brain swelling after stroke by about a third [26], prompting an ongoing search for a small molecule that inhibits AQP4. Such a treatment would complement rather than replace ChABC treatment because AQP4 inhibition increases the resistance to water flow across the BBB while ChABC treatment reduces the osmotic pressure driving that flow. Another recently proposed treatment for brain edema employs an osmotic transport device that is placed on the exposed cortex to draw water out of the tissue [21]. Chondroitinase ABC treatment would again complement this therapy because it would reduce the competing osmotic pressure that tends to hold water in the tissue.

The ICV route of administration was selected because it offered a combination of efficacy and clinical convenience. Intraparenchymal injection of ChABC in mice affects only a small region of the brain around the injection site and therefore may be inappropriate for treating more widespread edema [17]. Intravenous delivery is impractical because ChABC is too large to cross an intact BBB. A ventricular shunt is typically placed in patients with severe TBI to drain CSF as a first-line therapy to combat edema and control ICP [27]. This shunt can be used to administer therapeutic agents. Idursulfase, an enzyme with a similar substrate, successfully permeates brain tissue when administered to primates via ICV injection [28], suggesting that ICV delivery of ChABC would be similarly successful. In our study, an efficacious dose of ChABC was delivered via the ventricular space remote from the site of injury, suggesting that this strategy can be used to address global edema using ventricular shunts that are routinely placed as part of first-line therapy. However, the murine brain is much smaller than the human brain and the lissencephalic anatomy may allow easier access to the swollen cortex than the gyrencephalic anatomy of the human brain. Therefore, ICV delivery may be less effective in the human. Another important limitation of the current study is that treatment was administered within 5 min post-injury. The time to reach, stabilize and transport a severely brain injured patient creates an unavoidable delay of several hours between injury and treatment. Further investigation is necessary to fully understand how the timing of the therapeutic dose influences the time course of post-injury edema. Unfortunately, a thorough investigation of time course may require a different animal model. In humans, the brain is massive and is confined within the rigid cranial vault. This confinement creates positive feedback between edema, intracranial pressure and ischemia. This phenomenon is known as runaway intracranial hypertension and it strongly influences the course of post-traumatic edema in the days following a severe TBI. In mice, the brain is smaller and the skull is more flexible so this feedback effect is weaker. Therefore, the model presented here is appropriate to the study of post-traumatic edema but not for the study of subsequent runaway intracranial hypertension. Nevertheless, these preliminary results show that chondroitinase ABC can mitigate post-traumatic edema in vivo and this indicates that it may mitigate runaway intracranial hypertension in humans.

## Conclusions

This study demonstrated that ICV administration of ChABC eliminated almost half of the edema induced by CCI TBI in mice. Chondroitinase ABC has been tested extensively in the mouse brain for other purposes (i.e. not for treating edema) without evidence of toxicity [14–17]. Therefore, we believe that ChABC may be a promising new therapy for addressing post-traumatic brain edema.
